# Assessing Biomarkers of Porcine Kidneys under Normothermic Machine Perfusion—Can We Gain Insight into a Marginal Organ?

**DOI:** 10.3390/ijms251910280

**Published:** 2024-09-24

**Authors:** Carla Steinhauser, Abdulbaki Yakac, Wenke Markgraf, Susanne Kromnik, Andreas Döcke, Philipp Talhofer, Christine Thiele, Hagen Malberg, Ulrich Sommer, Gustavo B. Baretton, Susanne Füssel, Christian Thomas, Juliane Putz

**Affiliations:** 1 Department of Urology, Faculty of Medicine, University Hospital Carl Gustav Carus, Technische Universität Dresden, D-01309 Dresden, Germany; 2 Institute of Biomedical Engineering, Technische Universität Dresden, D-01309 Dresden, Germany; 3 Institute of Pathology, Faculty of Medicine, University Hospital Carl Gustav Carus, Technische Universität Dresden, D-01309 Dresden, Germany

**Keywords:** reperfusion-injury, normothermic machine perfusion, kidney, biomarker, pig

## Abstract

To identify potentially transplantable organs in a pool of marginal kidneys, 33 porcine slaughterhouse kidneys were perfused for 4 h with whole blood. During the normothermic perfusion, plasma, urine, and tissue samples were taken. Several biomarkers for tubule injury, endothelial activation, and inflammatory response were evaluated for a potential correlation with macroscopic appearance, histology, and filtration activity. Generally, biomarker levels increased during perfusion. TLR-4, EDN-1, and NGAL were not associated with any classification. In contrast, a steeper increase in NAG and IL-6 in plasma correlated with a poor macroscopic appearance at 4 h, indicating a higher inflammatory response in the kidneys with worse macroscopy early on, potentially due to more damage at the tubules. Although long-term effects on the graft could not be assessed in this setting, early observation under machine perfusion with whole blood was feasible. It allowed the assessment of kidneys under conditions comparable to reperfusion. This setting could give surgeons further insight into the quality of marginal kidneys and an opportunity to pre-treat them.

## 1. Introduction

The survival and quality of life of patients with end-stage kidney disease (ESKD) are improved after kidney transplantation compared to dialysis [1,2,3]. However, transplantation is limited due to the availability of donated organs. Additionally, donated organs are often marginal, based on an increasing number of older donors with comorbidities [4]. Marginal kidneys are more prone to delayed graft function (DGF) and primary non-function (PNF) [5,6]. As physicians can only rely on donor data and the macroscopic appearance of the kidney, marginal organs are often discarded even though they might still be functional [7,8].

These kidneys are susceptible to prolonged cold ischemia; therefore, shortening the storage time on ice between removal and transplantation has the potential to reduce injury. Replacing static cold storage (SCS) time with hypothermic machine perfusion (HMP) has already been proven successful in reducing the incidence of DGF [9,10,11].

Still, to properly utilize marginal kidneys for transplantation with little risk to the recipient, a functional assessment giving insight into organ quality is necessary. Normothermic machine perfusion (NMP) is a potential method to fulfill this role. An NMP device allows the ex vivo perfusion of an organ with oxygenated perfusate or whole blood at body temperature. Using whole blood, NMP could closely simulate the physiological conditions of the reperfusion of the organ after transplantation ex vivo. The setting of NMP has the potential to recondition organs and to observe their performance [12,13,14,15].

As the settings differ, no consensual criteria predicting transplantability have been agreed upon. Nevertheless, several have been explored: a lower score of quality criteria using the parameters of a macroscopic evaluation, renal blood flow, and urine production was linked to better short- and long-term outcomes in a series of transplanted kidneys that were assessed during NMP by Hosgood et al. [16]. Markgraf et al. used an experimental assessment of the potential functionality showing that porcine kidneys could be grouped based on their ability to clear inulin from the perfusate during NMP [17].

With NMP as a tool, it is possible to provide information and more insight into the extent of invisible injury. Markers giving insight into the dimension of damage and the potential functionality would be invaluable to the deciding surgeon. So far, the search for reliable biomarkers for kidney injury predicting graft functionality during NMP with whole blood is still underway [18].

Our goal was to identify biomarkers that could possibly be used to distinguish transplantable kidneys from non-transplantable ones and give insights into the kind of damage so that medical staff has an extra tool for evaluation of marginal organs. For this purpose, the levels of the vasoconstrictor endothelin 1 (EDN-1); the immune receptor Toll-like receptor 4 (TLR-4); the pro-inflammatory cytokine interleukin 6 (IL-6); and the tubuli damage markers kidney injury marker 1 (KIM-1), neutrophil gelatinase associated lipocalin (NGAL), and N-acetyl-/β-glucosaminidase (NAG) during NMP were investigated.

The potent vasoconstrictor EDN-1 is produced in a multitude of renal cell types [19,20,21,22,23]. It binds to its receptors distributed within the kidney [24], where EDN-1 is required for the kidneys’ functionality [25]. TLR-4 is an immune receptor best known for recognizing bacterial components and binding endogenous danger-associated molecular patterns released during tissue injury such as heat shock proteins [26,27] or components of the extracellular matrix [28,29]. After activation by ligand-binding, TLR-4 induces pro-inflammatory signaling pathways, making it an important mediator in the sterile inflammatory response [30]. The high concentrations of IL-6 directly after transplantation and before rejection have been known for decades [31]. IL-6 is a pro-inflammatory cytokine that has been linked with exuberating kidney injury [32,33] and transplant rejection [34,35], making it an interesting marker for kidney injury during machine perfusion. Urinary NAG has been known as a biomarker for kidney injury [36] and for its association with poor patient outcomes for a long time [37,38,39]. NAG is an enzyme predominantly expressed in cells of the proximal tubulus [40] and an increased urinary excretion has been observed after ischemic injury [41]. KIM-1, also known as hepatitis A virus cellular receptor 1 (HAVCR-1), is a well-known biomarker for kidney injury. It is a transmembrane protein expressed mainly in the kidneys, specifically in the proximal tubules [42]. After being cleaved by metalloproteinases, the exodomain of the protein is released into the tubule lumen, which leads to its excretion into the urine [43]. This shedding process is accelerated after injury to kidney cells [44], making it easy to detect in the urine after injury. NGAL is expressed in the injured parts of the kidney after ischemia-reperfusion injury [45]. It is localized in the cilium of tubular epithelial cells and is released into the urine after the loss of the cilium after injury [46]. As ischemia severely damages tubules [47], NGAL is often used as an injury marker.

## 2. Results

### 2.1. Kidney Classification Based on Macroscopy and Remuzzi-Score Was Inhomogeneous

**Figure 1:**
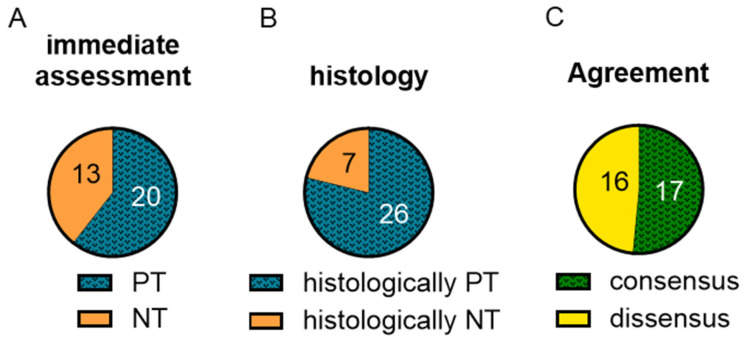
Kidneys were grouped into PT or NT based on the macroscopic assessment during NMP (**A**) and the histology, assessed by the Remuzzi score [48] (**B**). The agreement of the macroscopic assessment and the Remuzzi score was determined for each kidney (**C**).

The classification of the kidneys based on macroscopy resulted in 20 “potentially transplantable” (PT) and 13 “not transplantable” (NT) kidneys. (Figure 1A). Moreover, 26 kidneys scored three points or lower on the Remuzzi score. They were therefore considered histologically PT. Seven kidneys scored four points or higher and were therefore considered histologically NT (Figure 1B). The concordance between the macroscopy and the histology was only 51.5%. Within the concordance group of seventeen kidneys grouped into either PT or NT by both classifications (Figure 1C), fifteen were considered PT and two were considered NT by both classifications. Eleven histologically PT kidneys were assessed as NT by the macroscopic classification, whereas five histologically NT kidneys were assessed as PT.

### 2.2. pNAG and pIL-6 Correlated with the Macroscopic Clinical Classification under NMP

**Figure 2:**
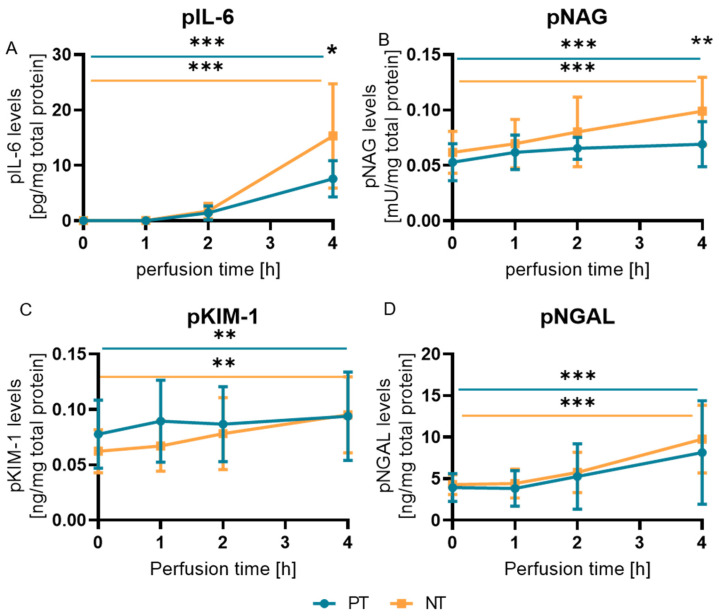
Kidneys were grouped into PT or NT based on their macroscopy during NMP. Plasma samples were taken from the perfusion circuit at 0 h, 1 h, 2 h, and 4 h. The plasma concentration of IL-6 (**A**), NAG (**B**), KIM-1 (**C**), and NGAL (**D**) was determined and normalized to the total protein concentration. Depicted is the mean (± SD). Statics were calculated for each group between 0 h and 4 h (paired analysis) and for each time point between the groups (unpaired analysis). * *p* < 0.05, ** *p* < 0.01, *** *p* < 0.001.

At the start and after 1 h of NMP, plasma IL-6 (pIL-6) was undetectable. An increase to about 1.5 pg/mL/total protein was measured after 2 h of NMP over all kidneys. After 4 h, pIL-6 levels were increased to 7.6 ± 3.2 pg/mL/total protein in the PT kidneys, compared to 15.3 ± 9.1 pg/mL/total protein in the NT kidneys (Figure 2A). NAG plasma (pNAG) concentration was also significantly higher (*p* = 0.0016) in NT kidneys at the end of NMP. While pNAG levels increased slightly in the PT kidneys from 0 h to 4 h (0.053 ± 0.016 to 0.069 ± 0.02 mU/mL/total protein; *p* = 0.0017), they increased at a greater degree in the NT group (0.062 ± 0.018 to 0.099 ± 0.029 mU/mL/total protein; *p* = 0.0007) (Figure 2B).

Despite the plasma KIM-1 levels (pKIM-1) and plasma NGAL (pNGAL) increasing during NMP, they did not differ significantly between the two groups (Figure 2C,D). While urinary marker levels increased overall during NMP, they did not differ between NT and PT kidneys (Figure S1).

No significant difference existed between the groups’ gene expression of the analyzed biomarker genes in the tissue. KIM-1 mRNA was not detectable at all. NGAL mRNA levels were detectable but did not change throughout NMP. EDN-1 mRNA levels increased, but the rise was only significant in NT kidneys. Both groups’ IL-6 and TLR-4 mRNA levels also increased significantly from 0 h to 4 h (Figure S2).

### 2.3. Biomarkers Did Not Reflect Histological Changes

Only 21% of the kidneys were considered histologically NT. Again, none of the plasma biomarkers could distinguish between kidneys with a low Remuzzi score and kidneys with a higher score. Gene expression was similar to the previously discussed results in the macroscopically classified kidneys. KIM-1-mRNA levels were undetectable in tissues and the relative expression of EDN-1, IL-6 and TLR-4 increased between 0 h and 4 h, but did not differ between the groups. The relative NGAL expression was higher in histologically NT kidneys at 0 h than in PT kidneys (0.0096 ± 0.0048 vs. 0.0154 ± 0.0066, *p* < 0.05) (Figure S3). However, this difference was no longer observable at the end of NMP. Biomarkers in plasma and urine tended to increase significantly during the duration of the perfusion. However, based on the histological classification they did not differ significantly between histologically NT and PT (Figure S4).

### 2.4. Functionality Did Not Correlate with Macroscopic or Histologic Assessment

Knowing the potential functionality of the kidneys before transplantation would allow a more accurate assessment of the kidneys’ performance. Consequently, the kidneys’ glomerular filtration rate (GFR) was assessed via their inulin clearance [17].

**Figure 3:**
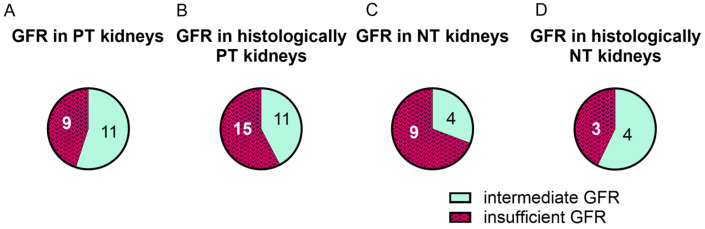
The potential functionality of kidneys grouped into PT and NT based on either their macroscopy during NMP (macroscopic assessment) (**A**,**C**) or their histology (**B**,**D**). GFR was assessed using the inulin clearance as a measurement for potential functionality. GFR < 2.04 mL/min/100 g was considered insufficient and 2.04 ≤ GFR ≤ 4.43 mL/min/100 g was considered intermediate [17].

Eleven of them had an intermediate GFR and nine had an insufficient GFR, whereas four NT kidneys had an intermediate GFR and nine NT kidneys had an insufficient GFR (Figure 3A,C). In the histologically PT kidneys, eleven had an intermediate GFR and fifteen had an insufficient GFR. Four of the histologically NT kidneys had an intermediate and three an insufficient GFR (Figure 3B,D). The intermediate and insufficient GFR functionality groups were comparatively evenly distributed between the NT and PT kidneys. No trend was observed to indicate that either PT or histologically PT kidneys were likelier to have an intermediate GFR than an insufficient GFR.

### 2.5. GFR in PT Kidneys Correlated with uIL-6

As NT kidneys would not be considered for transplantation, only PT kidneys were analyzed to find biomarkers correlating with the potential functionality. If found that conclusions concerning the functionality could be drawn during NMP. In general, all tested biomarkers increased during NMP. PT kidneys with insufficient GFR had significantly higher urinary Il-6 (uIL-6) levels in the 1 h urine sample than PT kidneys with intermediate GFR (0.33 ± 0.48 vs. 0.02 ± 0.04 pg/mL/total protein). No differences were observed in the plasma levels or the tissue gene expression of IL-6 in the PT kidneys with different GFR (Figures S5 and S6).

### 2.6. ROC Analysis of pIL-6, uIL-6 and pNAG Supported Performance Prediction

**Figure 4:**
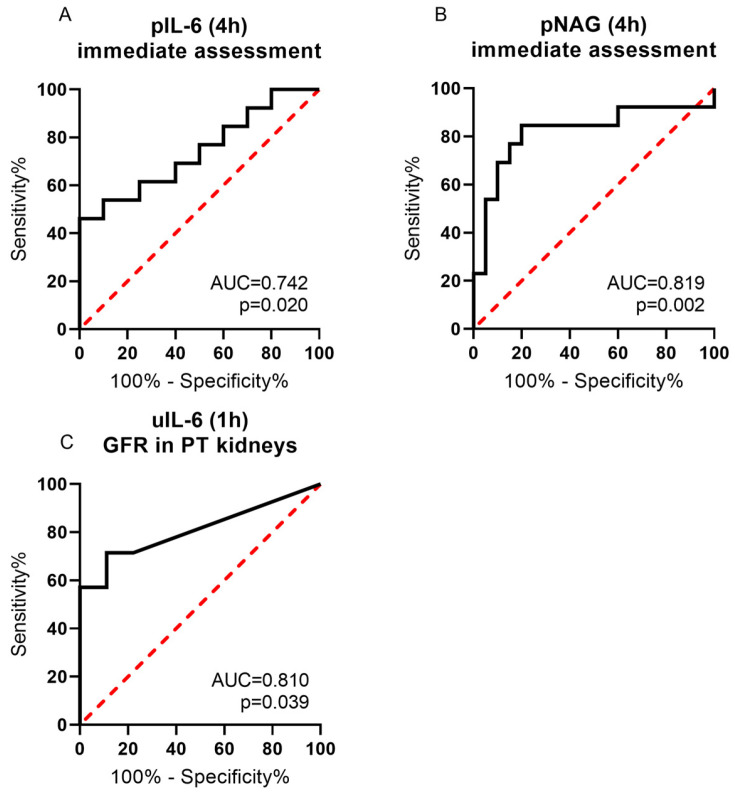
Prediction of potential functionality. ROC curves were calculated from the data sets of pIL-6 at 4 h, classified by macroscopy. (**A**) pNAG at 4 h, classified by macroscopy (**B**) and uIL-6 at 1 h, PT kidneys classified by functionality (**C**). Area under the curve (AUC) was also calculated. The red dashed line represents the line of no discrimination with an AUC of 0.5, the statistical result of a random guess.

ROC curves to predict kidney transplantability were calculated for the biomarkers that differed significantly between the groups. pIL-6 differed significantly between PT and NT kidneys at 4 h. The AUC was 0.742 with a sensitivity of 46.2% and a specificity of 100%, indicating a fair fit (Figure 4A). The optimal calculated cut-off was >15.73 pg/mL/total protein. pNAG also performed well in differentiating NT and PT kidneys with an AUC of 0.819 with a sensitivity of 84.6% and a specificity of 80% (Figure 4B). The calculated cut-off was >0.079 mU/mL/total protein. uIL-6 at 1 h performed similarly in PT kidneys grouped according to the GFR as determined by the inulin clearance. The AUC was 0.810, the sensitivity was 71.4%, and the specificity was 88.9% at the calculated optimal cut-off of >0.078 (Figure 4C).

## 3. Discussion

Several biomarkers were chosen to assess potential injuries that might occur during reperfusion. As such, EDN-1 was chosen to evaluate endothelial activation, KIM-1 and NGAL were chosen as markers of tubular injury and TLR-4 and IL-6 were chosen to investigate the potential activation of the immune system. The biomarkers will each be discussed in the following.

### 3.1. EDN-1 as a Biomarker

EDN-1 expression is increased both in chronic kidney disease [49] and after acute renal injury after ischemia-reperfusion (IRI) [50]. EDN-1 has also increased at mRNA- and protein levels after machine perfusion [51,52,53,54]. One group reported a correlation between excreted EDN-1 and the extent of kidney damage [55,56]. While we observed an upward trend in EDN-1 mRNA levels in the tissue, we could not observe a different expression in the more and less macroscopically or histologically damaged kidneys, even though one would expect a faster increase at the mRNA level. It is possible that the secreted EDN-1, a processed peptide [25], is quickly generated by processing the previously produced prepropeptide without immediately increasing EDN-1 transcription. Therefore, it seems that EDN-1-mRNA is not a suitable marker in this normothermic perfusion setting.

### 3.2. TLR-4 as a Biomarker

TLR-4 was elevated in deceased donor kidneys compared to those from living donors [57]. Additionally, loss-of-function mutations in the *TLR-4* gene were associated with better immediate graft function [57]. Furthermore, the deletion of TLR-4 or its downstream adapter molecule myD88 resulted in less ischemia injury than the wild type [58,59]. When comparing two different settings, TLR-4 correlated to increased ischemia-reperfusion injury in a machine perfusion setting [52,60]. Based on these data, we expected a higher expression of TLR-4 in the NT, histologically NT and insufficient GFR groups compared to their respective less injured group. While recent studies have shown that elevated TLR-4 correlates with the extent of transplant injury and its function, we were unable to link TLR-4 to impaired macroscopy, histology or functionality. It might be possible that 4 h NMP was not long enough to induce a strong TLR-4 response in the way it has been observed in transplanted organs. However, the overall increase in TLR-4 levels between 0 h and 4 h was indicative of the inflammatory response caused by the reperfusion. Hosgood et al. used a cytokine absorber to remove cytokines from the perfusate and found no increase in TLR-4 between the start and end of perfusion [61]. This might indicate that the TLR-4 increase is directly tied to the rapid rise in cytokines, such as IL-6.

### 3.3. IL-6 as a Biomarker

Hosgood et al. found increased urinary levels in kidneys with a longer warm ischemic time [55]. Another study described increased urinary IL-6 concentrations in cold storage compared to machine perfusion at 22 °C and to NMP [62]. We were also able to observe a fast increase in Il-6, both at the mRNA and protein level, after the start of reperfusion in all kidneys. The final plasma levels of IL-6 were significantly higher in the macroscopically NT kidneys compared to the PT kidneys. This indicates that the macroscopically more damaged kidneys were, in accordance with the aforementioned studies, injured in a way that promotes an unfavorable outcome. This negative effect from high IL-6 levels could be based on its effect on naive T cells. IL-6 prevents their differentiation into type 1 T helper cells and into regulatory T cells (T_reg_) while simultaneously promoting differentiation into type 17 and 2 T helper cells (T_h17_, T_h2_) [63,64,65,66]. This combination tilts the inflammatory response into something more harmful to the graft as T_h17_ are involved in the rejection reaction [67]. At the same time, fewer T_reg_ are available, which could down-regulate the immune response [68]. In fact, continuously high levels of T_reg_ are associated with better long-term graft survival [69]. Therefore, higher levels of IL-6 are at risk of impeding the graft. The macroscopic assessment aligned with high pIL-6 levels, relating the poor perfusion of the organ to a stronger immune response. Whether the early high levels of IL-6 observed in this study predict the long-term IL-6 expression and secretion remains to be elucidated. Additionally, it seems that using whole blood facilitates inflammation, so further studies on the graft quality while using anti-inflammatory agents would be of interest.

### 3.4. NAG as a Biomarker

In the machine perfusion setting, findings concerning NAG vary considerably. On one hand, higher NAG levels in the perfusate were correlated with an increased risk for DGF [70]. On the other hand, post-transplant plasma NAG levels did not differ between non-injured and injured kidneys [71]. Pool et al. found an increasing NAG level during HMP [72], whereas Venema et al. observed a decrease in uNAG during NMP [73]. NAG excretion might possibly be dependent on the varying perfusion settings. In this study, we surprisingly found pNAG, but not uNAG, to be increased in macroscopically NT kidneys. Due to its expression in epithelial tubulus cells and relatively large size preventing it from passing through the glomerular filter, we expected high levels of uNAG in damaged kidneys. The fact that NAG was present in the plasma might indicate that the glomerular filtration barrier was compromised. As tubular epithelial cells sustained damage, cell debris from necrotic cells might have blocked the tubular lumen and caused a backflow. Alternatively, the more extensive livid coloration of the NT kidneys is most likely due to local hemorrhage and microthrombi, which might have impaired the borders between the blood vessels and tubule.

### 3.5. KIM-1 as a Biomarker

We observed a significant rise of KIM-1 protein levels in urine and plasma, while PT and NT kidneys could not be distinguished. Yet, KIM-1-mRNA was undetectable in most samples at the beginning and the end of the perfusion most likely due to too few cDNA copies.

KIM-1 can aid in clearing apoptotic cell debris from the tubule lumen by binding phosphatidylserine and initiating phagocytosis [74]. The KIM-1-mediated phagocytosis also has an anti-inflammatory and anti-autoimmune property as it promotes T_reg_ [75]. Additionally, KIM-1 plays a role in the regeneration of the tubule after denudation [76,77]. This makes it difficult to discern if increased KIM-1-levels are inherently bad as one would expect from a biomarker, as it also has positive annotations. This might be one of the reasons why we were unable to distinguish between NT and PT kidneys. While KIM-1 might have been partly shed as a direct result of injury, its presence could also point to an attempt at “self-healing” within the kidney, possibly to the physiological setting of the NMP.

### 3.6. NGAL as a Biomarker

Higher levels of NGAL have been linked to unfavorable outcomes for the graft [78,79,80,81,82]. On the other hand, Banks et al. found no correlation between DGF and NGAL tissue staining [77]. Likewise, neither the risk of PNF or DGF nor graft survival correlated with the NGAL levels in the perfusate during machine perfusion [83]. Another study found that higher NGAL levels were associated with better graft function after transplantation [84]. This shows that the properties of NGAL are not necessarily as “black-and-white” as often assumed. The setting and the timing of the measurement could potentially have a great impact on the interpretation.

In our study, NGAL levels in plasma and urine did not differ, but mRNA levels at the start of perfusion were significantly higher in the NT kidneys, although this difference disappeared at the end of perfusion. In vitro, kidney cells have been found to secrete NGAL into the medium 1 h after hypoxia [41] indicating an early response. Kidneys may have started transcribing injury-associated genes during initial SCS, possibly resulting in the increased NGAL mRNA levels in the early stages of the perfusion. While both groups have sustained potential injuries under warm ischemic time (WIT) and cold ischemic time (CIT), this could indicate the NT kidneys were already more damaged during SCS. During the later stages of perfusion, both groups suffered from further injury upon reperfusion, leading to equal NGAL levels at the end of the perfusion. NGAL has also been shown to have protective qualities and limit kidney damage from oxidative stress after IRI [85]. So it is possible that NGAL, like KIM-1, can be both a marker for damage and an attempt of the kidney to limit damage.

### 3.7. Aptitude of the Classifications

As of now, the macroscopy of an organ is still the main decision factor for a transplant surgeon besides donor characteristics to accept a kidney for transplantation. However, is it enough to only assess marginal organs macroscopically, while damage that might restrict their later performance after transplantation is unclear? We showed that the biomarkers pIL-6 and pNAG were significantly increased in the macroscopically worse NT kidneys compared to the well-perfused PT kidneys. This indicates that the overall macroscopy was reflected on a molecular level. Whether these damages may or may not be associated with DGF or long-term graft survival, especially in marginal kidneys, will have to be assessed.

Evaluating the histology with the Remuzzi score allows for a standardized, quantifiable assessment, but is time-intensive and requires a specialized pathologist. However, none of the tested biomarkers was associated with increased histological changes. Overall, there was little difference between histologically NT and PT kidneys, and no kidneys scored higher than six points out of a possible twelve, with most histologically NT kidneys scoring four points. Additionally, only a small part of the kidney can be assessed which limits the results. This may be especially important in slaughterhouse kidneys as the resulting kidneys may be younger and therefore healthier compared to donated human kidneys. The Remuzzi score includes fibrosis, a pathologic manifestation of chronic inflammation caused by a repeated wound healing process [86]. As the overall time between the time of death of the pig and the end of kidney perfusion is less than a day, injuries to the kidneys occurring during SCS or NMP will not have resulted in fibrosis. As a conclusion, this classification of histology is not suitable to evaluate changes during NMP. This is also reflected in the fact that no injury marker correlated with the evaluated histology during NMP. While looking at the tubular atrophy alone, we also found no difference. So, the primary histology is more suited to screen for pre-existing histologic changes but not for evaluating changes during NMP, as it does not suitably reflect acute injury inflicted to the graft during NMP.

The inulin clearance was used to assess the filtration activity of the kidney during the NMP. Inulin, while not metabolized in the body, can be used to determine the GFR accurately. It can differentiate between perfused porcine kidneys from laboratory pigs, with less injured kidneys starting the filtration immediately [17]. Our study’s kidneys appeared too injured to reach full filtration performance within the 4 h of NMP. As a model for marginal kidneys, which are more susceptible to DGF [5,6] it may be possible that the perfused kidneys could not filtrate the inulin during NMP at a satisfactory rate, but would have recovered later.

### 3.8. NMP Setting

Autologous whole blood is rarely used in NMP research, which may make it difficult to compare or transfer results to other studies. However, erythrocyte-based perfusates are routinely used and have shown promising results [13,56,87,88]. In comparison to erythrocyte-based solutions, reperfusion with whole blood has been used as a close approximation of an experimental replacement for transplantation [89,90]. It is still a good approach in comparison to foreign blood, as there is no influence from immune cells due to the blood being genetically identical. Furthermore, autologous whole blood is more easily available in an experimental preclinical setting.

## 4. Materials and Methods

### 4.1. Setting

From a local slaughterhouse, 33 kidneys were obtained from several cross-breeds of German Landrace, German Large White, and Piétrain, weighing 105–150 kg. Autologous blood was collected and stabilized using 70 mL 0.2 M citrate buffer pH 5.0 containing 7500 units of heparin (Panpharma, Luitré-Dompierre, France) and 3 mL of 5% glucose solution (Braun, Melsungen, Germany) per 500 mL of blood. Kidneys were removed once the slaughterhouse process allowed, initially flushed with 40 mL isotonic NaCl solution (Braun) containing 400 units of heparin and then with 500 mL of histidine-tryptophan-ketoglutarat solution (Dr. Franz Köhler Chemie, Bensheim, Germany) containing 500 units of heparin. Stabilized blood and the flushed kidneys were stored bag-in-bag in ice-cold water.

### 4.2. Normothermic Machine Perfusion with Whole Blood

NMP was performed on an automated perfusion system, developed by the Institute for Biomedical Engineering (IBMT) (“nephroProtect”, project no. VP2904501KJ1, IBMT, Dresden, Germany) [17]. The device allowed control over blood temperature, flow, and pressure. Stabilized whole blood was oxygenated using a normoxic gas mix. Glucose was added to replenish blood sugar levels and Ringer’s solution (Fresenius Kabi, Bad Homburg, Germany) was added for compensation of lost volume. Perfusion pressure, blood flow, and resistance index were continuously monitored. Markgraf et al. previously described detailed instructions for perfusion circuit set-up and handling [17].

### 4.3. Macroscopic Classification of Kidneys

As of now, macroscopy of an organ is still the main decision factor for a transplant surgeon besides donor characteristics to accept a kidney for transplantation. During perfusion, a medical professional evaluated kidneys based on their overall appearance, grouping them into PT and NT. Kidneys with a homogenous or predominantly homogenous perfusion after 4 h of perfusion were considered PT.

### 4.4. Histological Classification of Kidneys

Evaluating the histology with the Remuzzi score allows for a standardized, quantifiable assessment, but is time-intensive in practice and requires a specialized pathologist. Biopsies were taken at the end of NMP from one pole of the kidney. They were fixated in formalin and embedded in paraffin. Hematoxylin–eosin staining was performed on 2 µm thick embedded sections. A pathologist scored the glomerular sclerosis, tubular atrophy, interstitial fibrosis, and the arterial/arteriolar narrowing and calculated the Remuzzi score (possible score: 0–12) without knowing the clinical classification [48]. Kidneys scoring a 0–3 were considered histologically PT, and kidneys with a score ≥ 4 were considered histologically NT.

### 4.5. Functional Classification of the Kidneys

The potential functionality of the kidneys was assessed via inulin clearance as described by Markgraf et al. [17]. Briefly, bolus inulin (10 µg/1 kg bodyweight of donor pig) (Sigma-Aldrich, Steinheim, Germany) was administered to the perfusing blood. The concentration of the remaining inulin in the blood was measured at 0 min, 5 min, 10 min, 20 min, and every following 20 min. Inulin concentration and, consequently, inulin clearance were determined using a two-compartment model [17]. A glomerular filtration rate (GFR) of <2.04 mL/min/100 g was considered insufficient and a GFR of >4.43 mL/min/100 g was considered sufficient based on their findings. A GFR in between these cut-offs was considered intermediate functional.

### 4.6. Analysis of Markers in Blood and Urine

Blood samples were taken at 0 h, 1 h, 2 h, and 4 h after starting the NMP and centrifuged to obtain the plasma. Urine samples were collected for a full hour and taken afterwards at 1 h, 2 h, and 4 h if urine was produced. Using ELISA-Kits, the concentrations of the potential markers KIM-1, NAG (both Bioassay Technology Laboratory, Shanghai, China), NGAL (MyBioSource, San Diego, CA, USA), and IL-6 (Cloud-Clone Corp., Katy, TX, USA) were determined. Total protein concentration was measured by bicinchoninic acid assay (Thermo Fisher Scientific, Darmstadt, Germany). Measured marker concentrations were normalized to total protein concentration. To differentiate them in this work, plasma biomarkers are written with the prefix “p”, and urinary biomarkers are written with “u” (e.g., pIL-6, uIL-6).

### 4.7. Gene Expression in Biopsies

To analyze the mRNA levels, biopsies were taken at the start and end of NMP from the kidney’s pole and preserved in RNAlater (Sigma-Aldrich, Steinheim, Germany) until they could be frozen and stored at −80 °C. Tissue was ground up and lysed in QIAzol (Qiagen, Hilden, Germany). RNA was extracted using a lipid tissue kit (Qiagen) and transcribed into cDNA using the reverse transcriptase Superscript III (Thermo Fisher Scientific), both following the manufacturer’s instructions. The cDNA of EDN-1, HAVCR-1 (hepatitis virus A cellular receptor 1, gene of KIM-1), IL-6, LCN2 (lipocalin 2, gene of NGAL), and TLR-4 was quantified by quantitative real-time PCR (qPCR) using Taqman assays (Table 1). PCR was run according to the manufacturer’s recommendations. The geometrical mean of GAPDH and RPL19 levels was utilized for normalization of the qPCR data.

### 4.8. Statistical Analysis

Statistical analysis was carried out using Prism 9.0 (GraphPad Software, La Jolla, CA, USA). Normality was tested for using the D’Agostino and Pearson K2 test. For unpaired non-normally distributed data, Mann–Whitney analysis was used. Unpaired normally distributed data were compared using unpaired *t*-test. For paired non-normally distributed data, Wilcoxon analysis was used. Paired normally distributed data were compared using paired *t*-test. Cut-offs for the receiver operated characteristic (ROC) curve analyses were calculated with the Youden Index. The area under the curve (AUC) of the ROC curves were also calculated for the prediction of potential transplantability.

## 5. Conclusions

Overall, biomarkers indicative of damage or inflammation in a porcine kidney under NMP with autologous whole blood increased during NMP of four hours. This may reflect the ischemia-reperfusion injury associated with inflammation and cell death in the affected tissue [91,92]. As reperfusion injury is expected after CIT, it might benefit the kidney to receive the ischemia-reperfusion injury outside the recipient’s body and not flood the recipient with cytokines and injury markers. Instead, the full extent of ischemia-reperfusion injury could be settled ex vivo during NMP, using the perfusate to flush out the cytokines, cell debris, and injury markers from the organ before transplantation, potentially negotiating part of the patient’s burden.

For this future research, the results will need validation in a transplantation model to accurately assess the post-transplant functionality of the kidneys. Finally, the transferability of the porcine biomarkers into a human setting will need to be tested in existing perfusion settings for human procedures.

## Figures and Tables

**Table 1:** Assay-ID numbers for the Taqman assays for the porcine genes EDN-1, GADH, HAVCR-1, IL-6, LCN2. RPL19, and TLR-4. All from Thermo Fisher Scientific.

Gene	Gene Abbreviation	Assay-ID
Endothelin 1	EDN-1	Ss03392453_m1
Glyceraldehyde-3-phosphate dehydrogenase	GAPDH	Ss03375629_u1
Hepatitis A virus cellular receptor 1	HAVCR-1 (KIM-1)	Ss04245599_m1
Interleukin 6	IL-6	Ss03384604_u1
Lipocalin 2	LCN2 (NGAL)	Ss04327246_m1
Ribosomal protein L19	RPL19	Ss03375624_g1
Toll-like receptor 4	TLR-4	Ss03389780_m1

## Data Availability

Data set available on request from the authors.

## Supplementary Materials

The following supporting information can be downloaded at https://www.mdpi.com/article/10.3390/ijms251910280/s1.
