# Supplementary material for: Assessing Biomarkers of Porcine Kidneys under Normothermic Machine Perfusion—Can We Gain Insight into a Marginal Organ?
Source: Int J Mol Sci. 2024 Sep 24;25(19):10280. doi: 10.3390/ijms251910280 (PMC11476884; doi:10.3390/ijms251910280)
Supplement: Supplementary file 1 [file ijms-25-10280-s001.zip › ijms-3125210-supplementary.pdf]

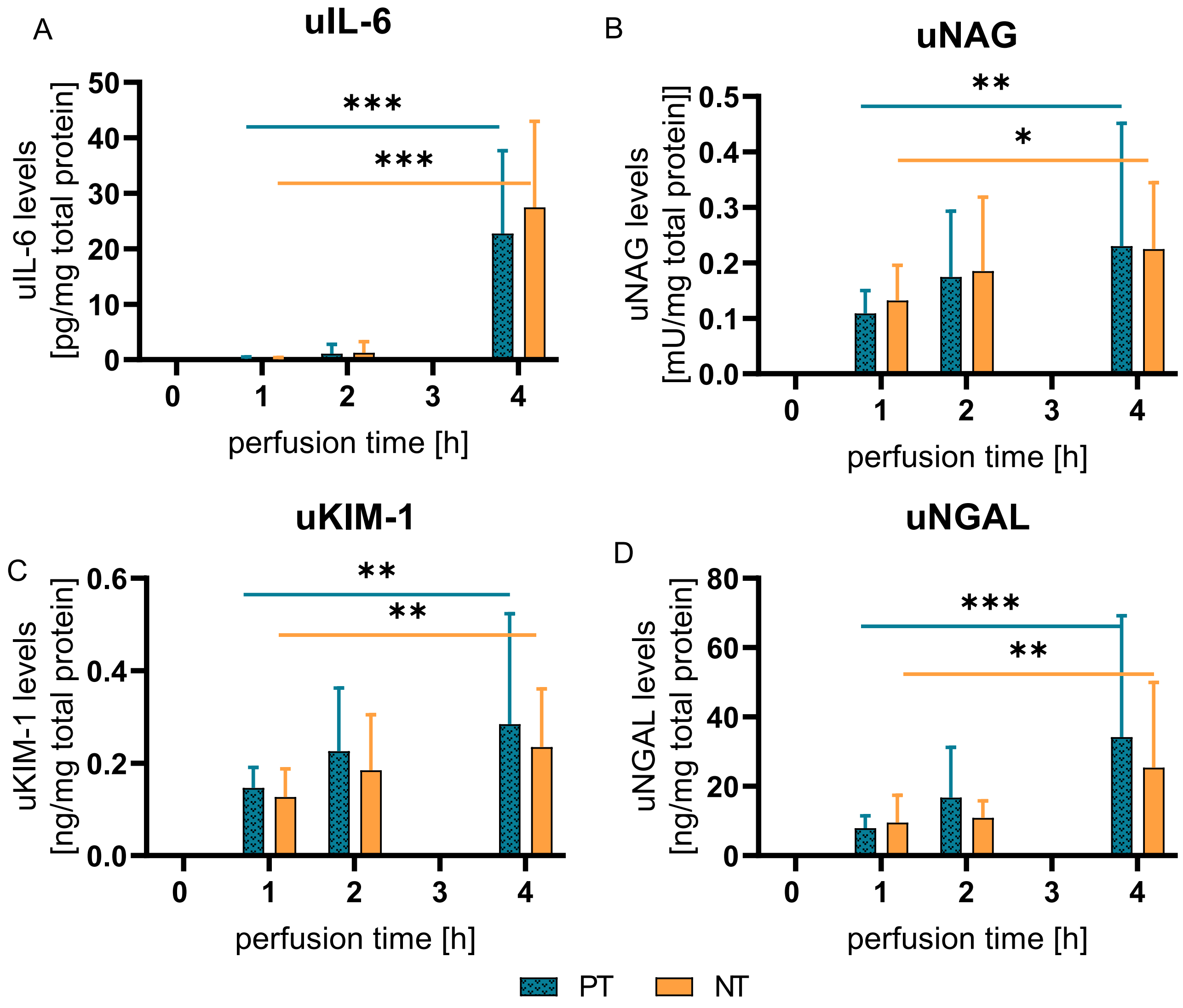

Figure S1: Kidneys were grouped into PT or NT based on their macroscopy during NMP. Urine was collected for 1 h each and taken at 0 h, 1 h, 2 h and 4 h. The urine concentration of IL-6 (A), NAG (B), KIM-1 (C) and NGAL (D) was determined and normalized to the total protein concentration. Depicted is the mean (SD). Statics were calculated for each group between 1 h and 4 h (paired analysis) as well as for each time point between the groups (unpaired analysis). *p<0.05, **p<0.01, ***p<0.001

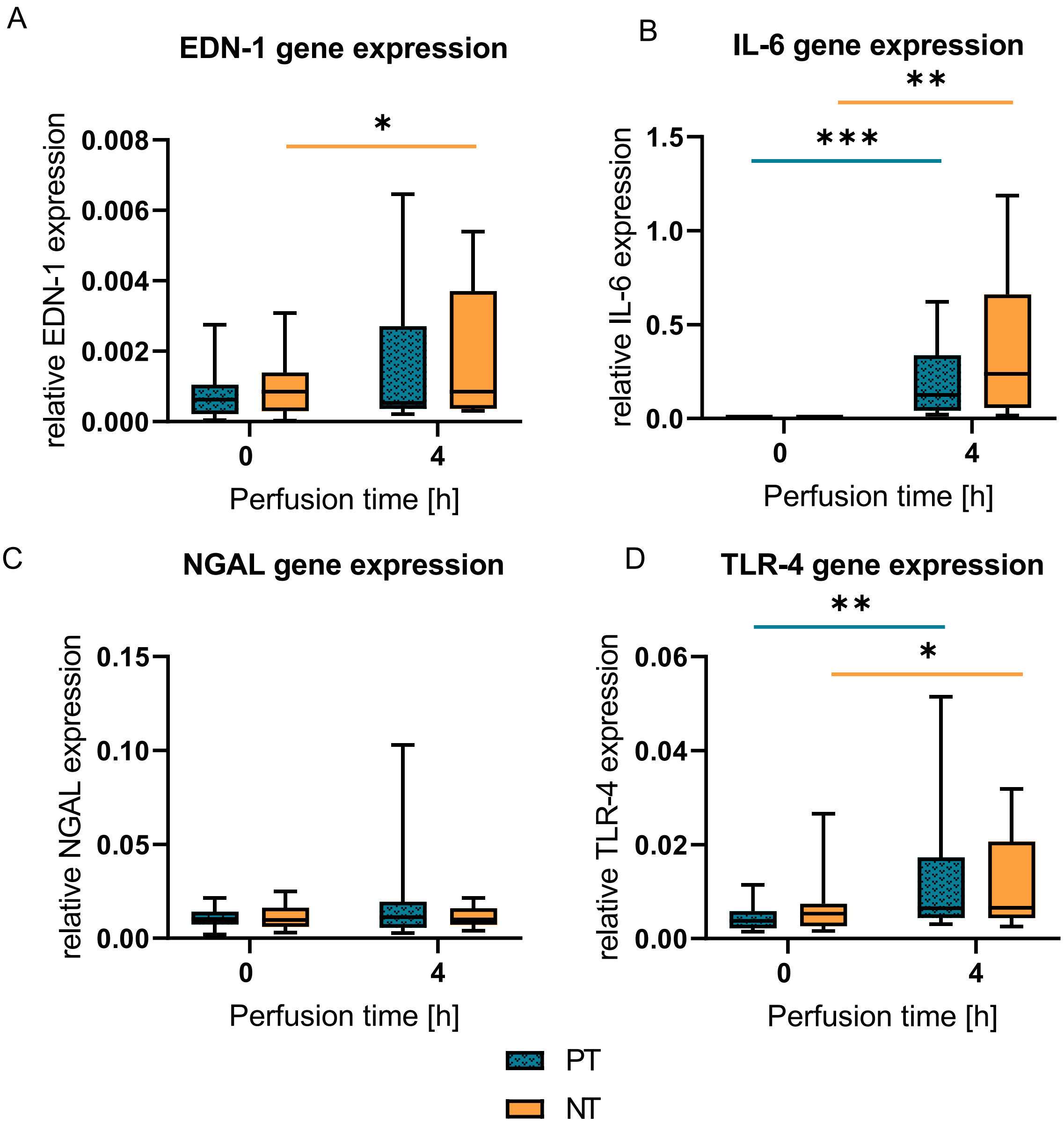

Figure S2: Kidneys were grouped into PT or NT based on their macroscopic assessment during NMP. Before the start and after the end of NMP a biopsy was taken and the tissue gene expression was analyzed using qPCR. Normalized expression levels of EDN-1 (A), IL-6 (B), NGAL(C) and TLR-4 (D) are shown. Box plots show 2nd and 3rd quartile with the median in the box, whiskers show minimum and maximum. Statics were calculated for each group between 0 h and 4 h (paired analysis) as well as for each time point between the groups (unpaired analysis). *p<0.05, **p<0.01, ***p<0.001

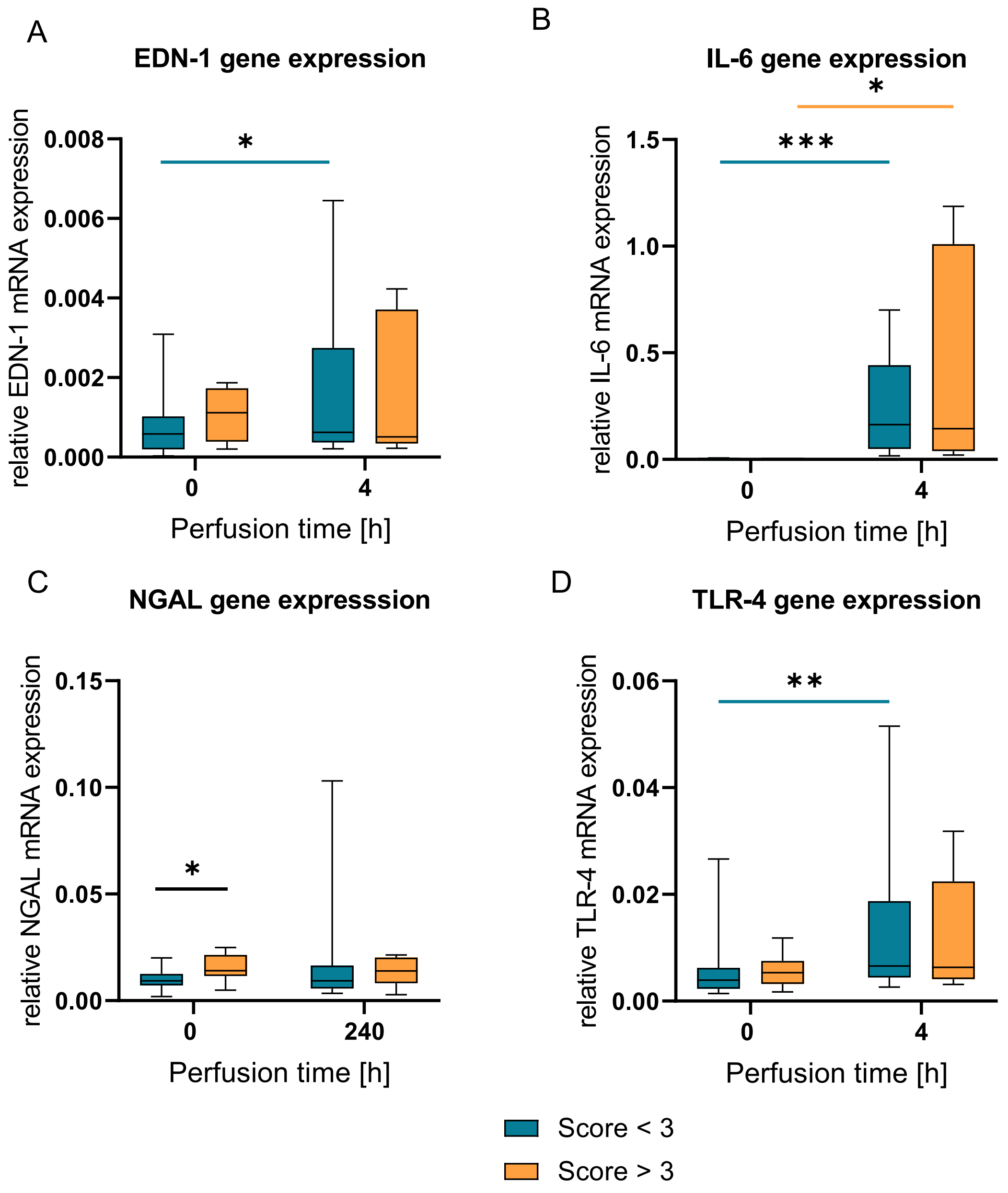

Figure S3: Kidneys were grouped into histologically PT or NT based on the Remuzzi score. Before the start and after the end of NMP a biopsy was taken and the gene expression was analyzed using qPCR. Normalized expression levels of EDN-1 (A), IL-6 (B), NGAL (C) and TLR-4 (D) are shown. Box plots show 2nd and 3rd quartile with the median in the box, whiskers show minimum and maximum. Statics were calculated for each group between 0 h and 4 h (paired analysis) as well as for each time point between the groups (unpaired analysis). *p<0.05, **p<0.01, ***p<0.001

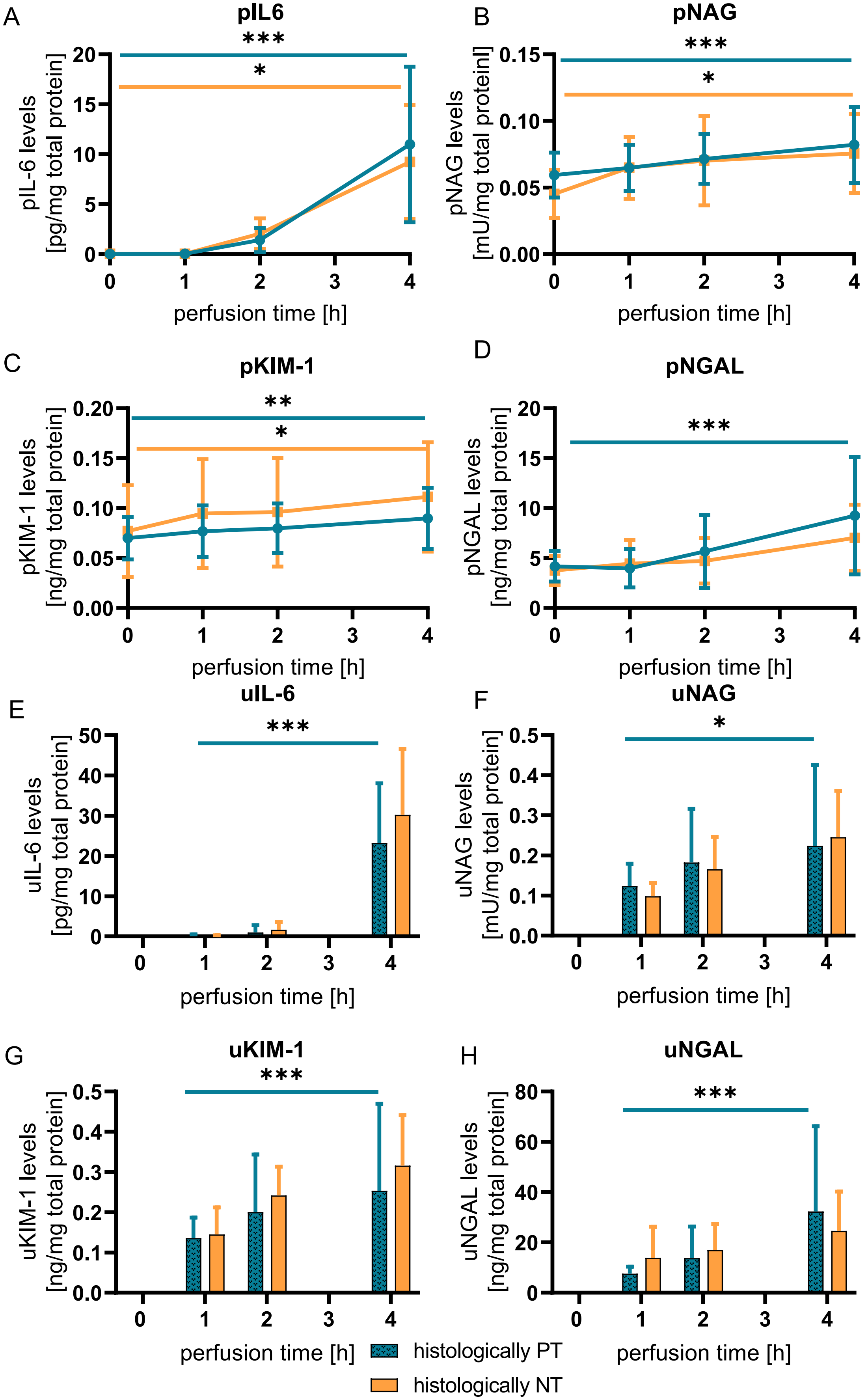

Figure S4: Kidneys were grouped into histologically PT or NT based on the Remuzzi score. Plasma samples were taken at 0 h, 1 h, 2 h and 4 h from the perfusate circuit, 1 h urine collections were taken at 1h, 2h and 4h each. The levels of pIL-6 (A), pNAG (B), pKIM-1 (C), pNGAL (D), uIL-6 (E), uNAG (F), uKIM-1 (G) and uNGAL (H) was determined and normalized to the total protein concentration. Mean (SD) was plotted. Statics were calculated for each group between 0 h/1 h and 4 h (paired analysis) as well as for each time point between the groups (unpaired analysis). *p<0.05, **p<0.01, ***p<0.001

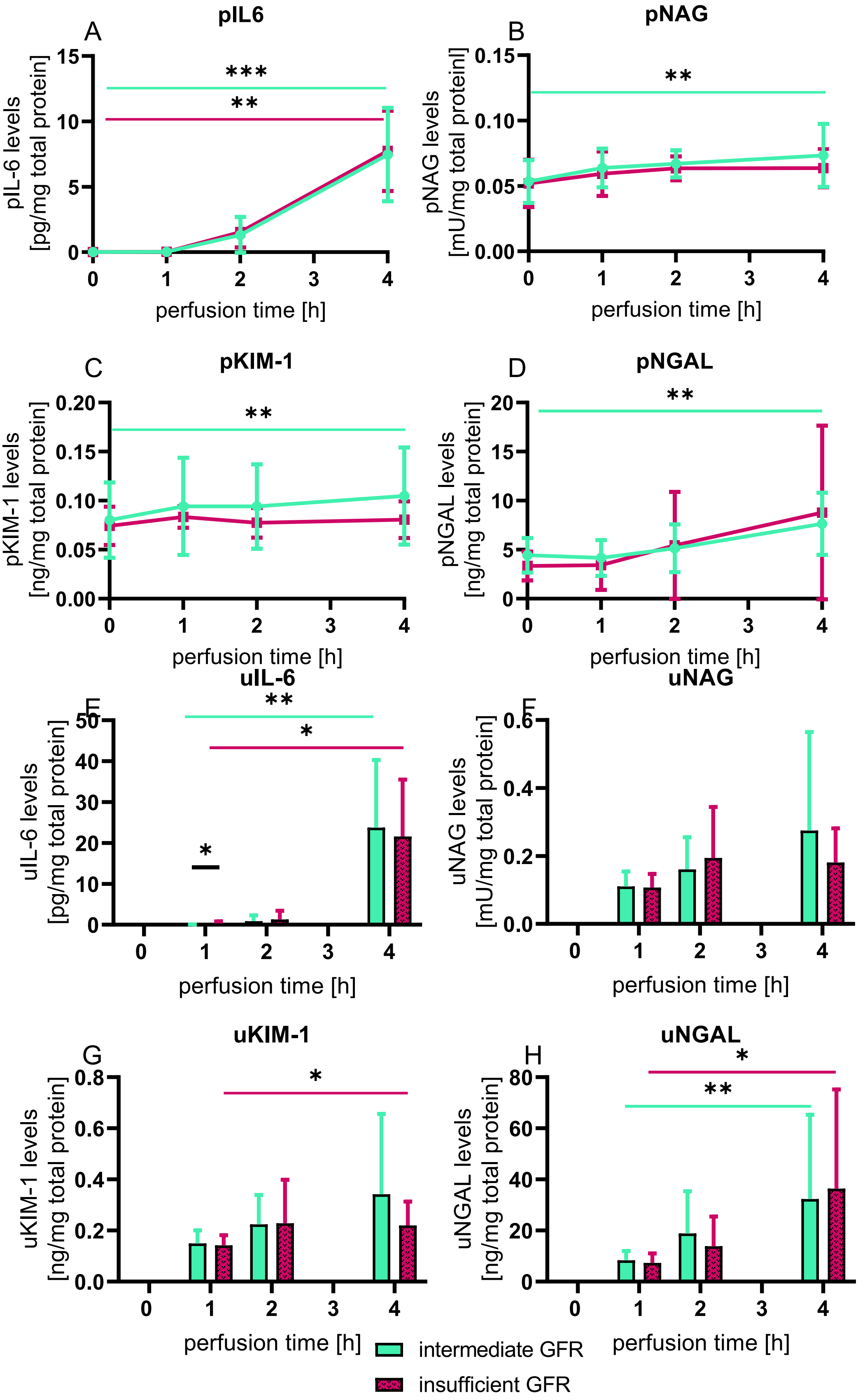

Figure S5: Kidneys that were considered "transplantable" by the macroscopic assessment were grouped into intermediate and insufficient GFR based on inulin clearance. Plasma samples were taken at 0 h, 1 h, 2 h and 4 h from the perfusate circuit, 1 h urine collections were taken at 1 h, 2 h and 4 h each. The levels of pIL-6 (A), pNAG (B), pKIM-1 (C), pNGAL (D), uIL-6 (E), uNAG (F), uKIM-1 (G) and uNGAL (H) was determined and normalized to the total protein concentration. The mean (SD) was plotted. Statics were calculated for each group between 0 h/1 h and 4 h (paired analysis) as well as for each time point between the groups (unpaired analysis). *p<0.05, **p<0.01, ***p<0.001

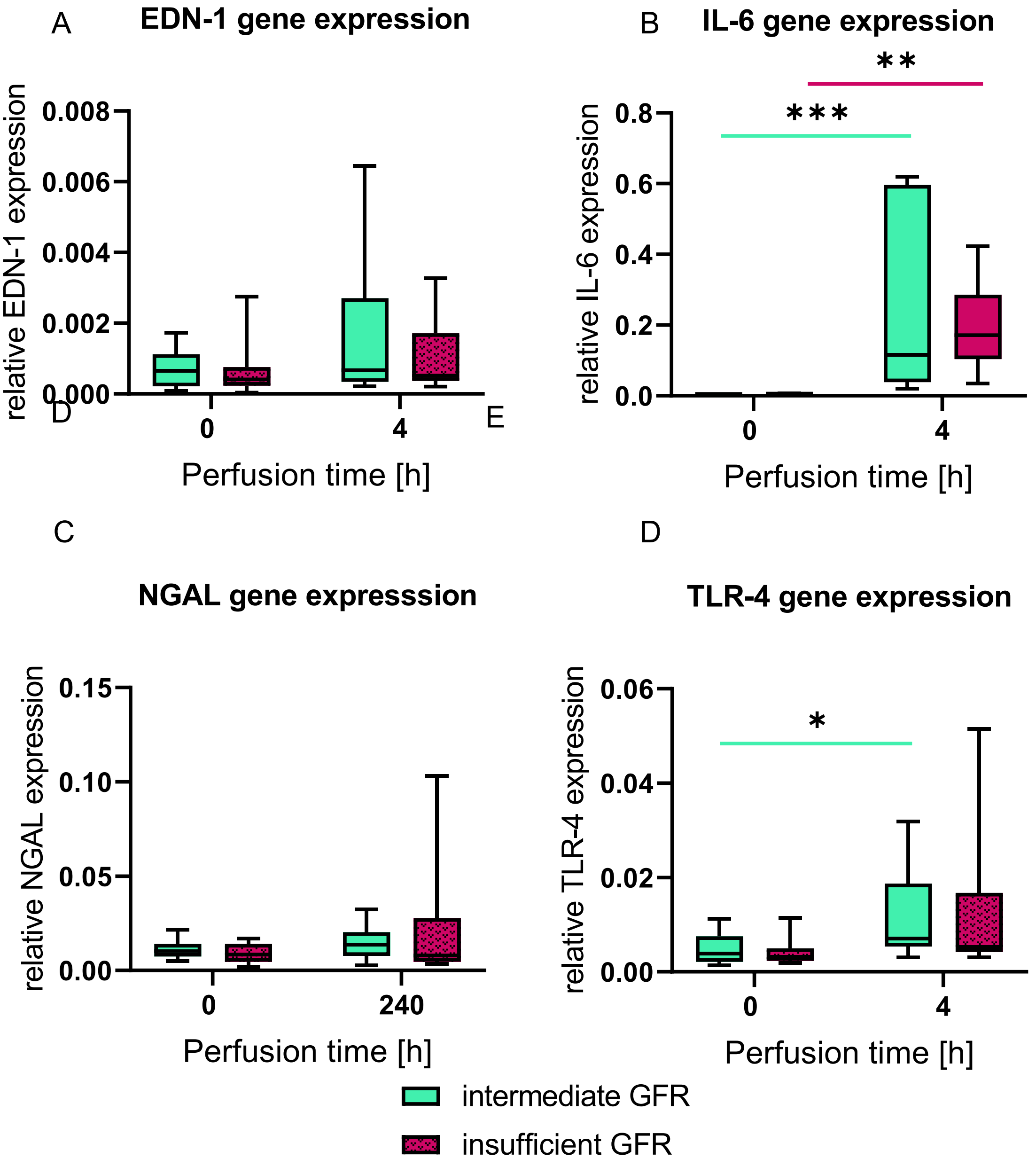

Figure S6: Kidneys that were considered PT by the macroscopic assessment were grouped into intermediate and insufficient GFR based on inulin clearance. Before the start and after the end of NMP a biopsy was taken and the gene expression was analyzed using qPCR. Normalized expression levels of EDN-1 (A), IL-6 (B), NGAL (C) and TLR-4 (D) are shown. Box plots show 2nd and 3rd quartile with the median in the box, whiskers show minimum and maximum. Statics were calculated for each group between 0 h and 4 h (paired analysis) as well as for each time point between the groups (unpaired analysis). *$p<0.05$, **$p<0.01$, ***$p<0.001$
